# Surface-Mediated Atomic Geometry and Decoupled States in Short Chains on a Si(553)–Au Surface

**DOI:** 10.1021/acs.jpclett.5c03189

**Published:** 2026-02-10

**Authors:** Tomasz Kwapiński, Mariusz Krawiec, Mieczysław Jałochowski

**Affiliations:** Institute of Physics, 49686 Maria Curie-Sklodowska University in Lublin, 20-031 Lublin, Poland

## Abstract

We demonstrate how the substrate fundamentally shapes atomic-scale systems on its surface and enables determination of their atomic geometry. Scanning tunneling microscopy and spectroscopy (STM/STS) measurements, supported by density functional theory and tight-binding calculations, reveal that even single adatoms exhibit a convolution with the substrate, leading to significant energy-level renormalization and a redistribution of their spectral weight beyond the energy-gapped region. In few-atom chains, this interaction drives surface-mediated dimerization, modifying both the geometry and electronic density of states, including the appearance of decoupled states. We show that, by explicitly incorporating the substrate’s electronic properties, one can not only reliably determine the atomic geometry and positions of adsorbed atoms, allowing for a proper interpretation of STM topography images, but also classify and assign the origin of the STS spectral peaks. Moreover, our findings challenge the common substrate d*I*/d*V* subtraction methods and the assumption that STM conductance peaks directly reflect molecular states, an interpretation that is not valid for gapped or semiconducting surfaces.

## Graphical abstract

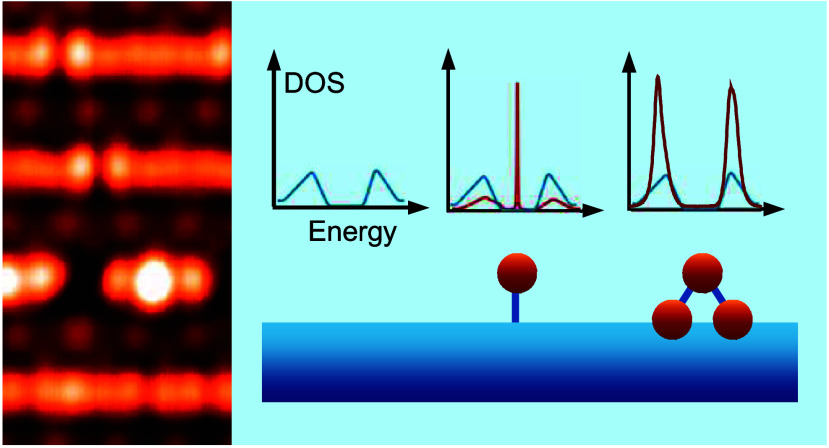

Individual atoms, dimers, or atomic chains positioned or fabricated on a surface represent quantum objects, exhibiting unique physical phenomena due to the confinement of electron movement to a low dimension. Examples include spin-charge separation,^1,2^ Majorana topological states,^3,4^ charge-density waves,^5–7^ and distinctive solid-state phases, such as time crystals.^8,9^ Such atomic-scale structures are typically fabricated on metallic or semiconducting surfaces and examined using scanning tunneling microscopy (STM), where the analysis of spectroscopic data is closely linked to the electronic properties of the studied systems, and the resulting topographic maps reveal their spatial atomic arrangement. This direct correspondence, however, is valid only for highly ordered atomic assemblies adsorbed on flat substrates with a constant or slowly varying spectral density function. In contrast, the topographic images corresponding to few-atom clusters or short chains are considerably more difficult to interpret. Due to symmetry breaking, it becomes challenging to determine how many atoms are present and what geometry they adopt, especially since STM topography often displays them as relatively uniform smeared bright blobs. This issue is particularly pronounced on non-metallic substrates, such as semiconducting surfaces with an energy gap or substrates exhibiting surface states or van Hove singularities. Therefore, developing a method that enables a reliable interpretation of STM topography and an accurate determination of the geometric arrangement of atoms in such surface-bound systems is of critical importance.

A prominent example of a system where few-atom chains of silicon can be routinely fabricated is the vicinal Si surface, which is stabilized by Au chains on terraces. Notable cases include Si(335)–Au,^10^ Si(557)–Au,^11^ Si(11 11 13)–Au,^12^ and Si(553)–Au.^13^ Among these, the Si(553)–Au surface has been the most extensively studied, both experimentally^14–20^ and theoretically.^17,21–23^ The atomic structure of this surface consists of step-edge Si chains with localized or weakly dispersed states^20,21,24,25^ and double Au chains in the middle of the terraces.^19^ During the formation of Si chains and surface ordering, the expanding domains of this chain lead to local mismatches and defects within the 1D structure. As a result, short Si edge chains may emerge on the Si(553)–Au surface; these systems serve as representative examples in our study allowing us to investigate their atomic geometry and electronic properties. An example of a topographic image of the Si(553)–Au surface, which includes a short Si chain, is shown in Figure 1 of the Supporting Information.

Our study focuses on the role of the substrate in determining the atomic arrangement as well as the electronic properties of single atoms and short silicon chains located at the step edges of Si(553)–Au terraces. The primary objective of our work is to demonstrate that investigations of atomic-scale surface systems using scanning tunneling microscopy and spectroscopy (STM/STS), combined with theoretical modeling, are sufficient to unambiguously determine their true atomic structure. We provide evidence that a reliable interpretation of experimental results requires explicit consideration of the spectral function, specifically the density of states (DOS) of the unperturbed, clean substrate. This aspect is often neglected, particularly for gapped substrates, which may lead to misinterpretation of the spectral peaks. Moreover, our findings confirm that the commonly used normalization of the tunneling current derivatives, achieved by subtracting d*I*/d*V* of the metallic substrate from that of single atoms, molecules, and one-dimensional (1D) nanostructures on substrates, such as Cu(110),^26^ superconducting Pb(100),^27^ Ag(111) single crystal,^28^ Cu_2_N/Cu­(100),^29^ Ru(0001),^30^ and Cu(111),^31^ is ineffective for the non-metallic substrates discussed in this letter.

In our work, we aim to address this issue by complementing STM/STS investigations of defected atomic chains on Si(553)–Au with theoretical calculations. To develop effective models for chains composed of only a few atoms, density functional theory (DFT) and tight-binding (TB) calculations were combined with three experimentally determined characteristics of the studied systems: the topography images of these structures, the local DOS of the terrace, and the differential conductance measured along these chains. Details of the experimental setup as well as the DFT and TB calculations are provided in the Supporting Information.

*Terrace and Single-Step Edge Atom*. Figure 1a presents a STM topographic image showing a small fragment of a long Si chain (at the bottom), the terrace, and a single Si atom that is separated by defects from two long chains on the left and right sides. A structural model of ideal non-defected Si(553)–Au, overlaid on this image, serves to identify the positions of neighboring atoms. In the ideal configuration, the step-edge atoms (brown balls) form long one-dimensional chains, but in the topographic image this ideal chain is disrupted by defects. The brown edge Si atom, located inside the small black rectangle in Figure 1a, is bound to two other atoms in the same terrace plane. This single Si atom possesses one free dangling bond and, within the framework of the TB model, can be effectively regarded as a site characterized by an on-site electron energy coupled with neighboring atoms.

**Figure 1:**
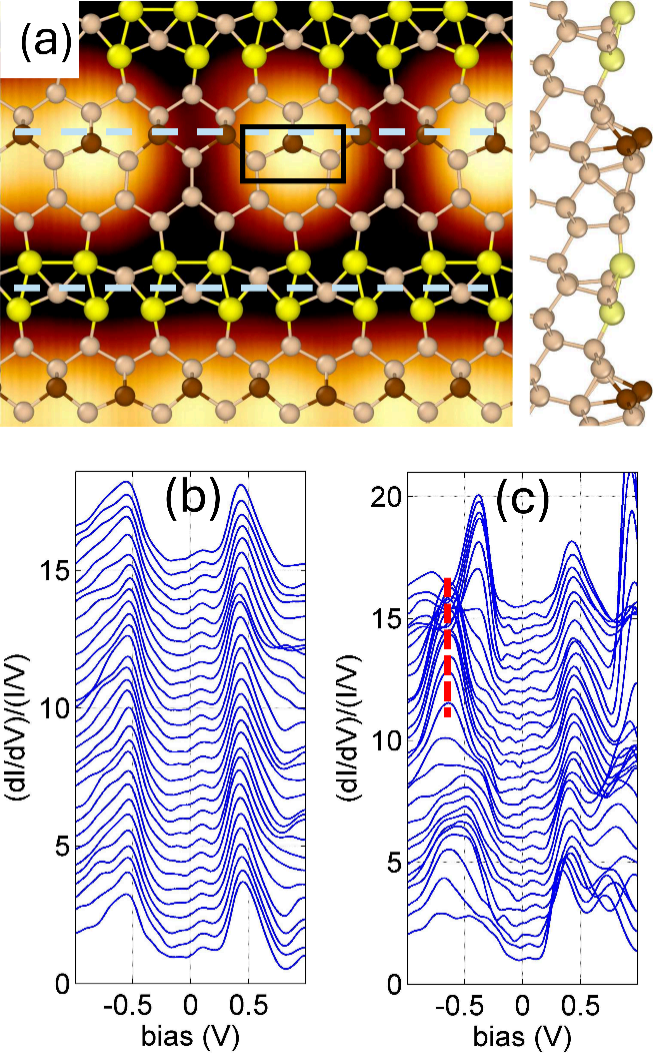
STM image of the Si(553)–Au surface featuring an isolated Si atom and superimposed structural model. (a) 2.9 × 2.3 nm topographic image (*U*_bias_ = −1 V and *I* = 50 pA) with the structural model proposed in ref 22. Yellow spheres represent Au atoms; brown spheres represent step-edge Si atoms; and light brown spheres represent other Si atoms. A black rectangle outlines the single Si atom and its nearest neighbors within the same terrace. Gray dashed lines indicate the positions where *I*(*V*) measurements were taken at equidistances of 0.10 nm. (b) Set of normalized d*I*/d*V* spectra measured along the terrace. (c) Normalized d*I*/d*V* spectra measured along the Si edge atoms. The red dashed line highlights data recorded at the site of the single Si atom. The first bottom lines in panels b and c were recorded at the left edge of panel a.

The set of curves in Figure 1b shows the normalized STM conductance of the terrace along the lower dashed line depicted in panel a. These curves will be used to characterize the electronic properties of the surface and will serve as the substrate DOS in theoretical calculations. According to DFT calculations (for details, see the Supporting Information), the two peaks located at −0.55 and +0.45 eV originate from the p_*z*_ orbitals of the double hybridized Au chain in the middle of the terrace.^22,25^ Another set of normalized conductance data along the edge chain (upper dashed line in Figure 1a), shown in Figure 1c, reveals a strong and narrow peak, marked with a vertical dashed red line, at −0.75 eV corresponding to the single Si atom. However, the origin of the peak is not simply related to the edge atom on-site energy and requires thorough discussion.

*Theoretical Model of a Substrate with an Adatom*. We begin by analyzing a single atom located at the terrace edge, as shown in the central part of the topography image in Figure 1a, considering two models of its interaction with the substrate, schematically illustrated as insets in Figure 2. The theoretical local DOS is derived from the corresponding diagonal matrix elements of the retarded Green’s function. This function depends on the atom–surface hybridization elements, thereby directly incorporating information about the surface DOS. Note that, in the calculations, the surface DOS is modeled by a two-peak function depicted with a black dashed line and has the same shape as the experimentally determined spectra shown in Figure 1b.

**Figure 2:**
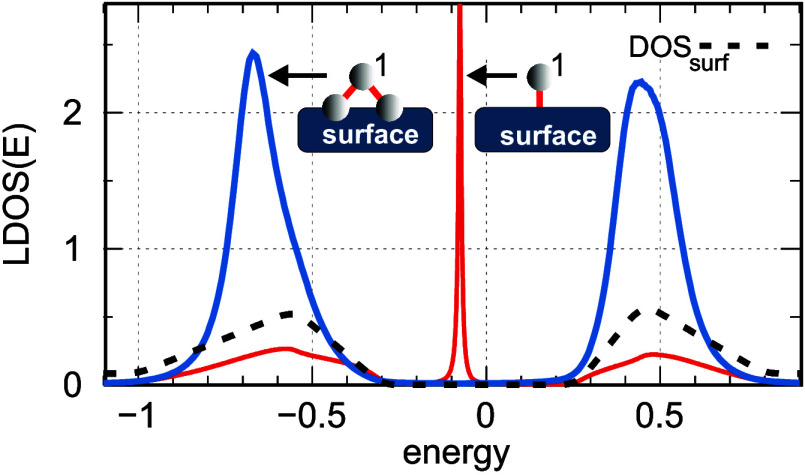
Red curve represents the local DOS of a single atomic site on the substrate. Blue curve depicts the LDOS for the model, where a single atomic site is coupled to two neighboring atoms placed directly on the substrate. The surface DOS used in the calculations is characterized by two peaks (black dashed curve) with an energy gap between them. The on-site energies of all atoms are set to ε_*i*_ = ε_0_ = −0.09; the coupling strengths between atoms are *t*_1_ = 0.4; and the surface–atom coupling is Γ = 0.05. All energies are expressed in eV units.

For the simplest model with a single atom (right inset, red curve), the spectral density at this site reveals a very sharp, nearly dispersionless bound state within the energy gap region of the substrate, along with two broad sideband peaks. These sideband peaks indicate a redistribution of the atomic spectral weight beyond the energy-gapped region, and they closely follow the substrate’s DOS structure, suggesting that they are linked to the surface’s electronic properties rather than the intrinsic atomic states of the edge atom. Additionally, the substrate’s energy gap is preserved in the local DOS, with only a single dispersionless bound state present within this region.

Since the shapes of the sideband peaks in the LDOS do not satisfactorily reproduce the STS spectra and no sharp localized states around the Fermi energy are observed experimentally, we consider in Figure 2 the results for an alternative system geometry using a more realistic extended model of a single edge atom on the substrate. In this model, the isolated edge atom is coupled to the substrate through its two neighboring atoms within the same terrace, while interactions with other atoms are incorporated as an effective coupling with the surface DOS. As shown in the figure, the local DOS of this atom (blue curve) is characterized by two sideband peaks separated by an energy gap. Moreover, the position of the left peak in the local DOS does not coincide with the maximum of the substrate DOS and is slightly shifted toward negative energies (compare the maxima of the black and blue lines). This shift results from the coexistence of two states with similar energies at about −0.55 eV (the molecular state of the atomic system and the states in the substrate DOS), leading to the renormalization of the atom’s spectral function. On the other hand, the position of the right peak in the local DOS function coincides with the maximum of the substrate DOS. Although the shape of this peak is significantly altered due to the interaction with the surface, deviating from a Lorentzian profile and exhibiting slight broadening, it aligns with our experimental results presented in Figure 1c. Thus, this system geometry more accurately reproduces the experimental data for a single atom at the edge of the surface.

Note that, in both system geometries discussed in Figure 2, the on-site atomic energy of the edge atom lies within the substrate’s energy gap. The absence of such states in the system described by a more realistic model (blue curve) is due to the presence of states decoupled from the substrate electrode, also referred to as dark states, in analogous to dark states in triple quantum-dot systems.^32,33^ These dark states arise when at least one of the molecular states of the atomic system does not involve all the atomic wave functions of the system, which occurs in our system.

*Short Atomic Chains*. The disruption of regular Si chains by local structural defects at the step edge or the mismatch between 1D domains with the 3× periodicity, which leads to the formation of short chains, requires thorough discussion. Figure 3a displays a STM topographic image showing two terraces of the Si(553)–Au surface with both short and long undisturbed chains, with the structural model of this ideal surface proposed in ref 22. The long chain (in the bottom part of panel a), which clearly exhibits 3× periodicity, serves as a reliable reference for identifying the edge Si atoms within the otherwise strongly smeared “blobs” in STM topographic images. As seen in the topography picture of the long chain, the slightly darker “blobs” contain two edge atoms that are indistinguishable in both STM topography and spectroscopy curves. This indicates that these atoms form a strongly coupled electronic state (atomic dimers). Correspondingly, the left short chain (along the L arrow), which is characterized by three bright blobs, can be identified as consisting of up to five Si edge atoms. The second short chain along this line is characterized by two bright blobs, indicating that it contains at least two atomic sites; however, the exact number of atoms in these structures and their geometry will be determined later through theoretical analysis. Note that, in the STM topographic images, both short chains exhibit spatial symmetry, as seen in Figure 3a and b. Therefore, the arrangement of the atomic sites should also preserve this symmetry.

**Figure 3:**
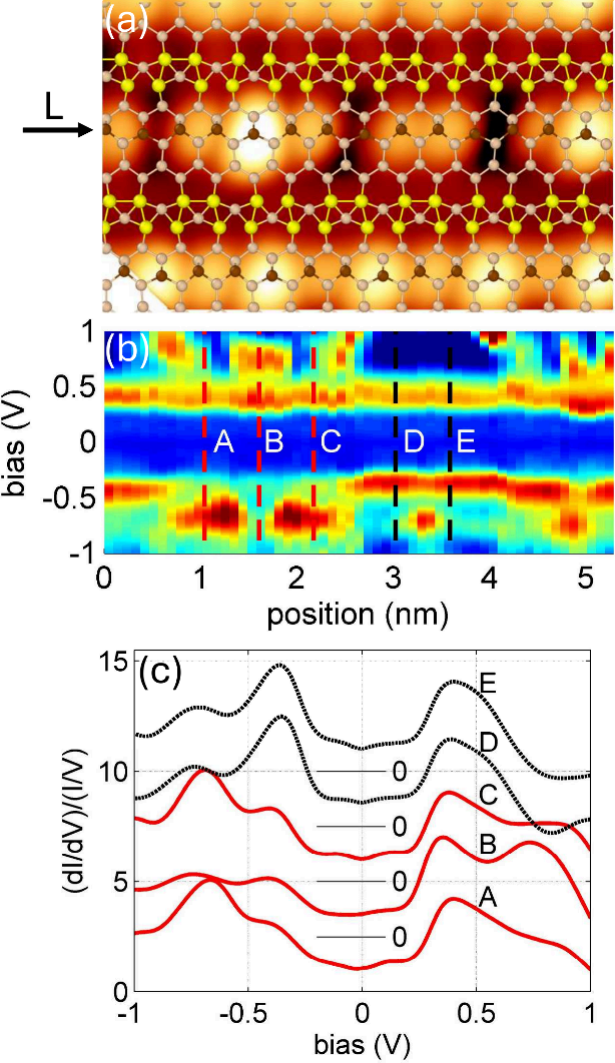
(a) Topographic STM image of the Si(553)–Au surface is presented with a superimposed structural model. The imaging conditions are a bias voltage of 0.75 V, a tunneling current of 50 pA, and a temperature of 77.6 K. Two short chains located at the terrace edge are marked with an arrow labeled L. The continuous Si chain at the bottom, which exhibits a periodicity of 3×, serves as a reference for aligning the theoretical structural model with the experimental data. (b) Normalized conductance map along the step edge of the short chains is shown. The colormap ranges from 0.5 units (dark blue) to 5.5 units (brown), indicating varying conductance levels. Vertical lines labeled A to E denote the positions of selected (d*I*/d*V*)/(*I*/*V*) curves, which are displayed in panel c.

In Figure 3c, selected STM normalized conductance curves measured on both short chains discussed above, labeled A–E, are presented. The curves A and C, corresponding to the end atoms in the longer chain, exhibit two states for positive voltages and two states for negative voltages. For each polarization, there is one prominent peak with high intensity and another significantly weaker one. On the other hand, the differential conductance in the middle of this chain, curve B, is also characterized by two peaks for positive voltage and two peaks for negative voltage, but their intensities differ upon reversing the sign of *U*_bias_. It leads to reversed contrast of topography images between middle and edge atoms for both negative and positive polarities. The normalized differential conductance of the second shorter chain, represented by curves D and E, shares almost the same shape, and correspondingly, the intensities of the topographic images varied in the same way. Both curves exhibit a single, very broad state (which could be a combination of two closely lying states) for positive voltage and two separate states (with significantly different intensities) for negative bias.

Based on these experimental results, effective geometrical models of both atomic structures were developed. In the calculations, each chain is described by the single-particle Hamiltonian with the nearest neighbor couplings between atomic states. For a regular chain with uniform atom–atom couplings and homogeneous on-site energies, it is possible to resolve this Hamiltonian analytically by means of Chebyshev polynomials of the second kind.^34–36^ Key factors of these models include the surface DOS, as determined from experimental data presented in Figure 1, the pairing of end atoms, and the shapes of experimental (d*I*/d*V*)/(*I*/*V*) curves shown in Figure 3c. Concerning the shorter chain case (on the right-hand side in Figure 3a), we initially ruled out the possibility that this chain consists of only two atoms, which might have been suggested by the topography due to the presence of two homogeneous and symmetric regions (“blobs”). However, the measured length of this chain indicates a structure composed of more than two atoms in a row. Moreover, theoretical calculations for a system of two coupled atoms on the surface, presented in the Supporting Information, show that, in this case, the local DOS does not reproduce the STS data, even qualitatively. Given the symmetrical topographic appearance of this short chain, we also excluded the possibility that it consists of three atoms forming a dimer plus a single Si atom, which together constitute the three-atom unit of an infinite Si(553)–Au edge chain. In that scenario, the topographic image should exhibit spatial asymmetry along the chain (like in the longer edge chain), which is not observed in this short system. Therefore, we considered alternative spatially symmetrical three-atom geometries (with a central edge atom coupled to two single atoms on its left and right, without dimerization) and performed the necessary computational calculations, which are also presented in the Supporting Information. It turns out that this geometry can also be excluded, as it does not accurately reproduce the experimental data. Consequently, for this chain, a four-atom model in the geometry of two coupled dimers shown in the inset of Figure 4 should be considered.

**Figure 4:**
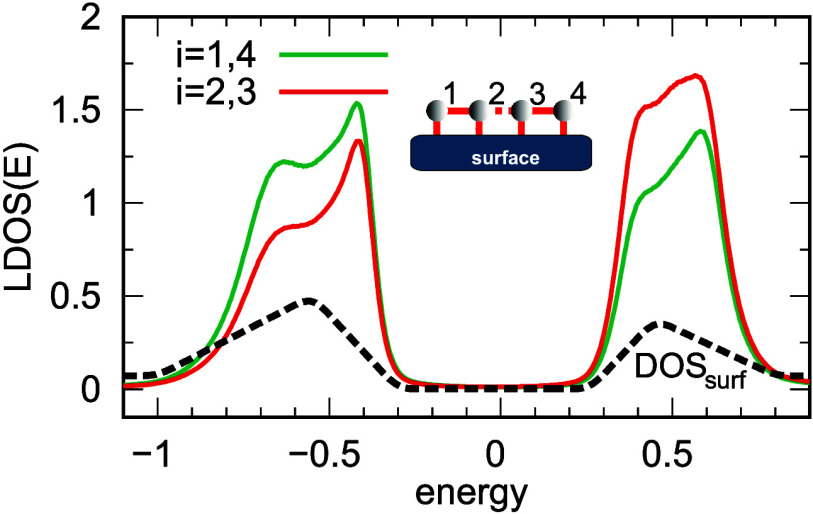
Local DOS for a system composed of *N* = 4 sites, for different atomic sites: *i* = 1 and 4, represented by the green curves, and *i* = 2 and 3, represented by the red curves. The inset schematically illustrates the system. The on-site energies are given by ε_1,4_ = 0.05 and ε_2,3_ = −0.1. The hopping integrals are *t*_1–2_ = *t*_3–4_ = 0.52, *t*_2–3_ = 0.11, and Γ_*i*_ = 0.04. The black dashed curve represents the surface two-peaked DOS that is used in the calculations.

Figure 4 presents the results for a system of two coupled dimerized atoms where all single-particle states in this atomic object remain within the energy gap of the substrate, whose DOS is represented by the black line. Notably, in this system, the local DOS at each atom features a broad peak at positive energies and a two-peak structure at negative energies. In the latter case, the state located at *E* = −0.4 exhibits a significantly higher intensity than the neighboring peak at −0.7. The differences in the behavior of the local DOS peaks for positive and negative bias voltages stem from the asymmetric shape of the substrate DOS peaks. Specifically, the peak for negative voltages is slightly broader compared to the peak for positive voltages (black line); see also Figure 1b. This indicates that the local DOS functions of the edge-chain atoms are influenced by the surface, redistributed beyond the surface energy gap, and represent a convolution of the molecular chain states with the substrate DOS peaks. As seen, the local DOS curves show good agreement with the differential conductance results obtained in the experiment (curves D and E in Figure 3), confirming the validity of the assumed geometry of the edge atom arrangement in this short chain.

The electronic properties of the second atomic chain with differential conductance characteristics marked by curves A–C in Figure 3 are analyzed in Figure 5. As before, the substrate spectral density is represented by the black curves, and in the upper panel, we consider a flat DOS function (wide-band approximation), while in the bottom panel, the surface DOS is described by the effective two-peaked function taken from the experimental results. The topography of this chain is characterized by three bright bolbs, which might initially suggest the presence of three sites in the structure. However, this interpretation was ruled out by analyzing and comparing the local DOS results for such a configuration to the STM spectroscopy data. The symmetric topographic image of the chain, considered together with the structural model of this ideal substrate, clearly indicates that the chain can be identified as consisting of five Si edge atoms. Therefore, this chain consists of *N* = 5 Si atoms, arranged with the linear geometry, as shown in the inset. Consequently, such a system contains two atoms that are strongly coupled to each other (atomic dimers) and weakly coupled to their neighboring central atom.

**Figure 5:**
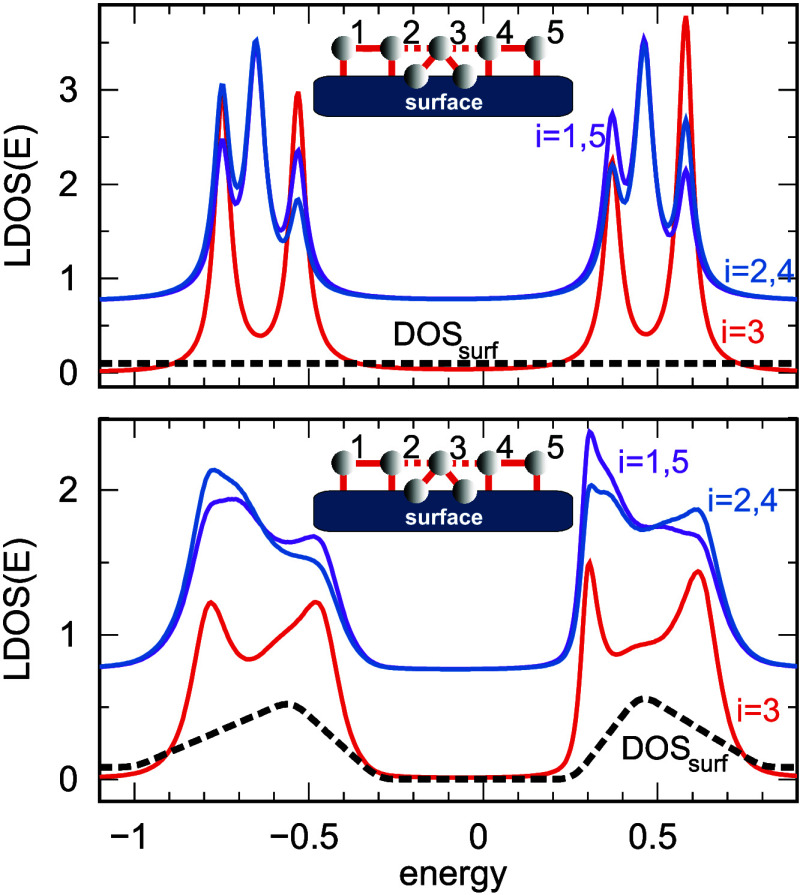
Local DOS for the system composed of *N* = 5 sites (schematically depicted in the inset) at atomic sites: *i* = 1 and 5, violet lines; *i* = 2 and 4, blue lines (shifted by +0.75 for better visualization); and *i* = 3, red lines. The black dashed lines represent the surface DOS used in the calculations: within the wide-band approximation (top panel) and for a two-peak DOS with an energy gap (bottom panel), with the on-site energies ε_1,2_ = ε_4,5_ = −0.1 and ε_3_ = −0.07 and the hopping integrals *t*_1–2_ = *t*_4–5_ = 0.55, *t*_2–3_ = *t*_3–4_ = 0.15, *t*_3_ = 0.38, and Γ_1,2,4,5_ = 0.04.

For the surface with a flat structureless DOS (top panel), each of the two strongly coupled atoms is characterized by three local DOS peaks at positive energies and three local DOS peaks at negative energies. At the same time, the middle atomic site (*i* = 3) is described by four regular local DOS peaks. The number of all LDOS maxima suggests the existence of six molecular states with different energies in our system. However, these results do not reflect our experimental data shown in Figure 3 (curves A, B, and C). In the bottom panel, we present the same results as in the upper panel but for a surface characterized by a real double-peaked DOS. In this case, the surface DOS and the double-atom segments (dimers) of the system (*i* = 1–2 or 4–5) determine the main structure of the local DOS, forming two broad sidebands at about +0.5 and −0.6. A more detailed analysis reveals a single dominant state at a negative energy at *E* = −0.8 (with a small feature in each curve at *E* = −0.4) and a double peak at positive energies. The local DOS of the middle atom has two states at negative energies and two distinct states at positive energies (red curve in the bottom panel). The absence of a mid-gap peak in local DOS is a signature of decoupled states. The results in the bottom panel in Figure 5 should be compared to the experimental data presented in Figure 3, and they indicate a good qualitative agreement between the theoretical and experimental data. It should be noted that the conductance signal originating from the substrate is typically removed by subtracting a background spectrum from the STS data.^26–31^ In our case, the isolated chain exhibits at least six molecular states (panel a). Subtracting the signal of the clean substrate from the experimental STS curves A–C in Figure 3c would fail to extract surface-independent information.

These findings highlight the significant role of the unperturbed substrate in the analysis of electronic states in the edge chain, where new spectral density peaks can appear or molecular states can vanish. It follows from theoretical considerations that all single-particle energy levels of the atomic states in the examined atomic systems lie within the substrate’s energy gap. However, the incorporation of Si atoms into the chains causes a transfer of spectral weight outside the energy gap, leading to the formation of a dimerized atomic structure. As a result, the local DOS peaks on each atom arise from the superposition of atomic states from neighboring atoms and interactions with substrate states present in the surface DOS. Therefore, attributing conductance peaks solely to atomic states or using background-removed spectra, as is often done in practice, is in general unjustified.

*Conclusion*. In this work, we demonstrate that the electronic band structure (DOS) of the substrate plays a fundamental role in determining the geometry and arrangement of atoms in the adsorbed structures on its surface. This is particularly important when analyzing STM topography images, as it is often challenging to determine whether a strong tunneling-current signal originates from a single atom, a dimer, or a group of atoms. We have shown that, by incorporation of the substrate’s DOS and employment of computational methods, one can definitively exclude certain atomic geometries and determine the actual atomic configuration in the studied system. The presence of the substrate also strongly influences the electronic properties of these structures. An accurate interpretation of STS and topographic data requires explicit consideration of the spectral density of the unperturbed, clean substrate. This is particularly crucial for semiconducting substrates that exhibit an energy gap or DOS singularities. By performing such an in-depth analysis, one can not only correctly assign the spectral features observed in STS but also account for states that are decoupled from the surface.

Both experimental observations and theoretical calculations demonstrate that even a single atom at the terrace edge undergoes strong renormalization of its energy levels, so that the features of the substrate’s DOS become clearly imprinted in its local DOS. Consistency between experimental results and theoretical DFT and TB calculations was achieved by recognizing the crucial role of the clean surface electronic properties. These findings challenge the conventional assumption that conductance peaks in STM measurements can be attributed solely to atomic or molecular states. Instead, the peaks arise from a convolution of the intrinsic molecular states of the chains and their interactions with the substrate’s electronic structure.

## Supplementary Material



## Notes

Experimental details, computational methods: DFT and TB, model parameters, and results for various atomic chain configurations (PDF)
