# Supplementary material for: Surface-Mediated Atomic Geometry and Decoupled States in Short Chains on a Si(553)–Au Surface
Source: J Phys Chem Lett. 2026 Feb 10;17(8):2191–6. doi: 10.1021/acs.jpclett.5c03189 (PMC12951569; doi:10.1021/acs.jpclett.5c03189)
Supplement: Supplementary file 1 [file jz5c03189_si_001.pdf]

Supporting Information

Surface-Mediated Atomic Geometry and Decoupled States in Short Chains on a Si(553)-Au Surface

Tomasz Kwapiński, Mariusz Krawiec, and Mieczysław Jałochowski*

Institute of Physics, Maria Curie-Sklodowska University in Lublin, Poland

E-mail: mieczyslaw.jalochowski@umcs.pl

I. Methods

Experiment

The experiments were conducted in an ultra-high vacuum system with a base pressure in the mid-10^{-11} mbar range. The system was equipped with a Reflection High-Energy Electron Diffraction (RHEED) diffractometer, an OMICRON LT STM/AFM apparatus, gold deposition sources, and a precise quartz microbalance sensor. N-type Si(553) samples with a specific resistivity of $0.002 \div 0.01$ $\Omega\cdot$ cm were cleaned by direct current (DC) flashing at approximately 1500 K. This process was monitored using RHEED until a clean surface free of SiC contamination was achieved. Subsequently, 0.48 ± 0.02 monolayers (ML) of gold (Au) were deposited onto the substrate at room temperature. Here, one ML is defined as half the

Si(111) surface atom density, equal to 7.84×10^{14} atoms/cm^2. During gold deposition, the pressure remained below 2×10^{-10}mbar. A well-ordered surface featuring one-dimensional structures along periodically arranged atomic steps was achieved by briefly heating the sample to 950 K for 2 seconds, followed by gradual cooling to room temperature over 3 minutes. To create short chains, no gases were intentionally introduced into the vacuum vessel; instead, these chains were identified among sparsely distributed imperfections between longer chains. STM topographic measurements in constant current mode were performed at 77.6 K. An example of such surface shows Fig.1. The normalized differential conductance curves, $(dI/dV)/(I/V)$, were numerically calculated from the experimental $I(V)$ data.

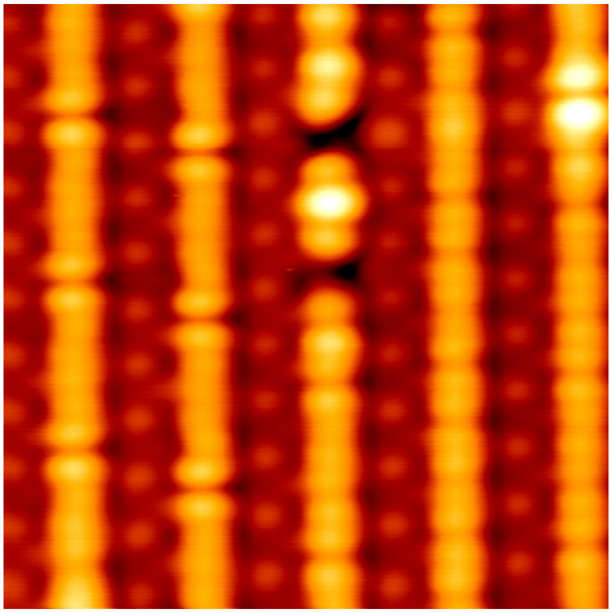

Figure 1: Topographic image of a 6 nm x 6 nm Si(553)-Au surface. The linear structures visible in the image are Si atomic chains exhibiting two different periodicities: x6 periodicity on the left side and x3 periodicity on the right side. Additionally, the dots with x2 periodicity correspond to a double Au chain. Short Si atomic chain is separated from long chains by two voids. This constant current image was obtained with a tunneling current of 50 pA and a sample bias of 0.25 V.

Computational methods

DFT: In this paper the DFT calculations have been performed using the projector augmented waves (PAW)[1] and the PBEsol correlation-exchange functional[2] available in VASP

(Vienna ab-initio simulation package).[3,4] In electronic structure calulations the HSEsol hybrid functional has been utilized.[5] A kinetic energy cutoff for the plane wave expansion of single particle wave functions was set to 340 eV. The Brillouin zone was sampled by $2\times4\times1$ Monkhorst-Pack k-points grid.[6] The convergence criterion for the total energy in the self-consistency cycle was chosen as 10^{-6} eV and the maximum force allowed on each atom during the geometry optimization was less than 0.01 eV/Å. The Si(553)-Au system has been built according to the structural model of Refs. 7,8, and consists of four Si double layers, passivated by H atoms at the bottom. Periodic replicas of the slab were separated by a vacuum gap of 19 Å. A surface supercell with 6×1 peridicity has been considered.

Below, we analyze the DOS of the Si(553) surface, theoretically calculated using DFT simulations. On a perfectly ordered Si(553)-Au surface, the atomic structure is defined by a 6×1 unit cell, with six pairs of Au atoms on the terrace. The local DOS is shown in Fig.2(a), while the key parameter—the averaged DOS of p_z orbitals—is presented in Fig.2(b). The calculations show that two peaks located at -0.55 eV and $+0.45$ eV originate from the double hybridized Au chain in the middle of the terrace.[8,9] Since electron tunneling techniques are primarily sensitive to p_z orbitals, it is justified to consider these states as the main contributors to the STM tunneling current. A similar averaging effect was observed in STM topographic images of terraces adjacent to disturbed Si chains at the step edge, acquired with a low gap voltage ($U_{bias}=0.2$ V).

Tight binding calculations: The system under consideration consists of atomic sites (either single atoms or a short linear chain) positioned on a surface. The model Hamiltonian of the system, written in the standard second-quantized form, can be expressed as the sum of the following components:

$$H = \sum_{i=1}^{N} \varepsilon_i a_i^\dagger a_i + \sum_{\vec{k}} \varepsilon_{\vec{k}} a_{\vec{k}}^\dagger a_{\vec{k}} + H_{coup} \qquad (1)$$

The first part describes the electron energies in the atomic chain of length N, where each

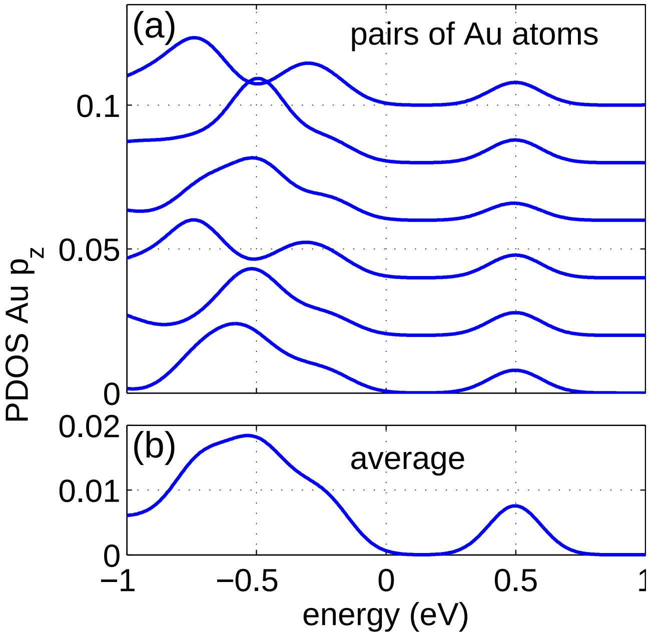

Figure 2: Theoretically obtained DOS of the double Au chains on the Si(553)-Au surface in the $6 \times a_{[1\bar{1}0]}$ unit cell, calculated for the rehybridized model.[8] (a) The DOS projected on p_z orbitals of six individual pairs of Au atoms. (b) Averaged contribution of the p_z orbitals to the DOS projected on the double Au chain obtained from curves in panel (a).

atomic site is characterized by the on-site electron energy, ε_i, while the second part corresponds to the surface electrons, $\varepsilon_{\vec{k}}$, characterized by wave vectors $\vec{k}$. The operators a_i ($a_i^\dagger$) annihilate (create) an electron at the i-th site of the chain ($i = 1, \ldots, N$), and $a_{\vec{k}}$ ($a_{\vec{k}}^\dagger$) are the corresponding annihilation (creation) operators for the surface. The third part of the Hamiltonian describes electron transitions along the chain, characterized by tunneling matrix elements $t_{i,i+1}$ between neighboring chain sites, and between the surface and chain sites, represented by hybridization elements $V_{i,\vec{k}}$. This Hamiltonian can be written as: $H_{coup} = \sum_{i=1}^{N-1}(t_{i,i+1}a_i^\dagger a_{i+1} + \sum_{\vec{k}} V_{i,\vec{k}} a_{\vec{k}}^\dagger a_i) + H.c.$

The local DOS function is related to the imaginary part of the retarded Green's function, providing insight into the electronic structure of the system.

$$LDOS_i(E) = -\frac{1}{\pi}\mathtt{Im}(G_{ii}^r(E^+))\,, \qquad (2)$$

where $G^r_{ii}(E)$ for the time-independent Hamiltonian satisfies the equation of motion: $EG^r_{ij}(E) = \langle [a_i, a_j^\dagger]_+ \rangle + \langle\langle [a_i, H]_-; a_j^\dagger \rangle\rangle_E$. Using this equation for N-site system the Green's function can be written in the matrix form: $\hat{G}^r \cdot \hat{A} = \mathbb{I}$, where $\mathbb{I}$ is the identity matrix and $\hat{A}$ is a square $N \times N$ complex array with the elements:

$$
\begin{aligned}
A_{i,j}(E) &= (E - \varepsilon_i)\delta_{i,j} - \Sigma_{i,j}(E) \\
&- t_{i,j+1}(\delta_{i,j+1} + \delta_{i+1,j}),
\end{aligned}
\tag{3}
$$

where $\Sigma_{i,j}(E) = \sum_{\vec{k}} V^*_{i,\vec{k}} V_{j,\vec{k}} (E^+ - \varepsilon_{\vec{k}})^{-1}$. The off-diagonal elements of $\Sigma_{ij}(E)$ rapidly vanish as they depend exponentially on i and j atomic distance[10,11] and in our calculations can be neglected. Diagonal terms of $\Sigma_{ij}(E)$ can be express in the following form $\Sigma(E) = \Lambda(E) - i\Gamma(E)/2$, where $\Gamma(E) = 2\pi \sum_{\vec{k}} |V_{\vec{k}}|^2 \delta(E - \varepsilon_{\vec{k}}) = 2\pi |V_{\vec{k}}|^2 DOS_{surf}(E)$. Here $DOS_{surf}(E)$ stands for energy dependent lead DOS. Note that both functions $\Lambda(E)$ and $\Gamma(E)$ are not independent on each other. They are related through the Hilbert transform: and the function $\Gamma(E)$, which depends on the substrate's DOS, determines the second function, $\Lambda(E)$. Together, they are responsible for the localized states appearing in the system. In the wide-band approximation, where the surface DOS is $\vec{k}$-independent, these functions are energy-independent: $\Lambda(E) = 0$ and $\Gamma(E) = \Gamma$. Under these conditions, localized states do not appear in the system.

However, when considering a specific surface DOS, such as that of two-dimensional materials with energy gaps or van Hove singularities, both $\Gamma(E)$ and $\Lambda(E)$ become energy-dependent. Localized states occur when $\Gamma(E)$ vanishes (i.e., outside the surface band or within the energy gap), which corresponds to the condition where $E - \varepsilon_0 - \Lambda(E)$ reaches its minimum value. In this scenario, the function $\Lambda(E)$ renormalizes the on-site energy position, leading to dispersionless states outside the substrate DOS.[12,13] For certain surface DOS profiles, such as rectangular or elliptic DOS, these functions can be obtained analytically. By knowing both $\Gamma(E)$ and $\Lambda(E)$ functions, one can explicitly determine all matrix

elements of $\hat{A}$ (Eq. 3). The retarded Green's functions can then be obtained by inverting this matrix: $G^r_{ii}(\varepsilon) = (\hat{A}^{-1})_{ii} = \texttt{cof}\hat{A}_{ii}/\det\hat{A}$, where $\mathrm{cof}(\hat{A}_{ii})$ denotes the cofactor (algebraic complement) of the matrix element $\hat{A}_{ii}$, and $\det(\hat{A})$ represents the determinant of the matrix $\hat{A}$. For a regular chain with uniform atom-atom couplings and homogeneous on-site energies, these functions can be expressed analytically for arbitrary N using Chebyshev polynomials of the second kind.[14–16] In the general case (for arbitrary on-site energies in the chain and beyond the wide band approximation), we obtain the local DOS along the chain for a given surface DOS numerically.

In the calculations it is necessary to determine how to effectively describe the DOS of the substrate, to which the short atomic chains will be coupled. For a regular substrate structure, the energy-band dispersion, $DOS(E)$, described by a TB Hamiltonian, can be expressed as $DOS(E) = \sum_n \int \frac{d\vec{k}}{(2\pi)^2}\delta[E - \varepsilon_n(\vec{k})]$, where the summation over n pertains to the energy bands in the Brillouin zone with energy dispersion $\varepsilon_n(\vec{k})$. In two-dimensional (2D) tight-binding lattices (e.g., square, triangular, honeycomb, Lieb, Kagome), the energy dispersion can be derived analytically and is expressed in terms of elliptic integrals. For example, in a 2D rectangular lattice, the surface dispersion relation is characterized by a van Hove logarithmic singularity in the middle of the band. Similarly, for the honeycomb lattice, the DOS exhibits two van Hove singularities with a local minimum in the center of the band. However, in our case, the surface is not flat and it consists of two types of atoms (Si and Au), thus it cannot be treated as an ideal 2D structure.

In our calculations, we consider the zero-temperature limit. Energies are expressed in units where $\Gamma_0 = 1$, with Γ_0 defined as $\Gamma_0 = 2\pi V^2_{\vec{k}}/w$, for a flat, $\vec{k}$-independent lead DOS of width w. The energy reference point is set at the chemical potential of the surface, $E_F = 0$.

II. Other geometries of short chains

In this section, we theoretically analyze the electronic properties of few-atom systems that may exist on the vicinal Si(553)-Au surface using the TB model supported by DFT and experimental data. Our goal is to optimally adjust the effective parameters describing these systems and to assess the feasibility of specific atomic geometries in real atomic chains. The substrate is characterized by a DOS function corresponding to the experimental measurements shown in Fig. 1b of the main text and confirmed by the DFT calculations (Fig. 2). The effective surface DOS used in the calculations is depicted in all theoretical plots as a solid black line with two peaks at energies $E = -0.55$ and $E = +0.45$, and an energy gap between them. Our primary focus is on the local DOS associated with the atoms forming the short two- and three-atom chains.

Two coupled sites at the surface: First, we analyze the system consisting of an atomic dimer placed on the surface, as shown in Fig. 3. The top panel presents the local DOS as a function of energy for different values of single-particle energy levels $\varepsilon_1 = \varepsilon_2$ (since the system is symmetric, the local DOS is the same for both atoms). Such two coupled sites is characterized by the presence of two molecular states, meaning that the local DOS function should exhibit two peaks. When the single-particle energies $\varepsilon_{1/2}$ lie outside the substrate's energy gap, the local DOS structure indeed shows two distinct peaks (for $\varepsilon_{1/2} < -0.3$ and $\varepsilon_{1/2} > 0.1$). Notably, molecular states that appear within the energy gap or far from the surface DOS peaks are nearly non-dispersive (having high intensity and being very narrow). It is worth noting that in the STM measurements, we do not observe non-dispersive states with very high intensity, thus such values of the parameters are invalid. As can be observed, the local DOS curves for the on-site energies in the range of -0.5 to $+0.1$ (i.e. inside the energy gap region) exhibit a somewhat different character, depicted as blue curves in the top panel. Firstly, the local DOS states have moderate intensity and are relatively broad. Additionally, the local DOS structure clearly reveals the effects of atomic interactions with the complex substrate DOS. Consequently, multiple local DOS peaks are observed, often with

inflection points, which more closely resemble the differential conductance curves measured in the experiment. Therefore, the appropriate range of energy level positions for Si edge atoms should correspond to the region of blue curves which is reasonable range considering isolated Si atoms.

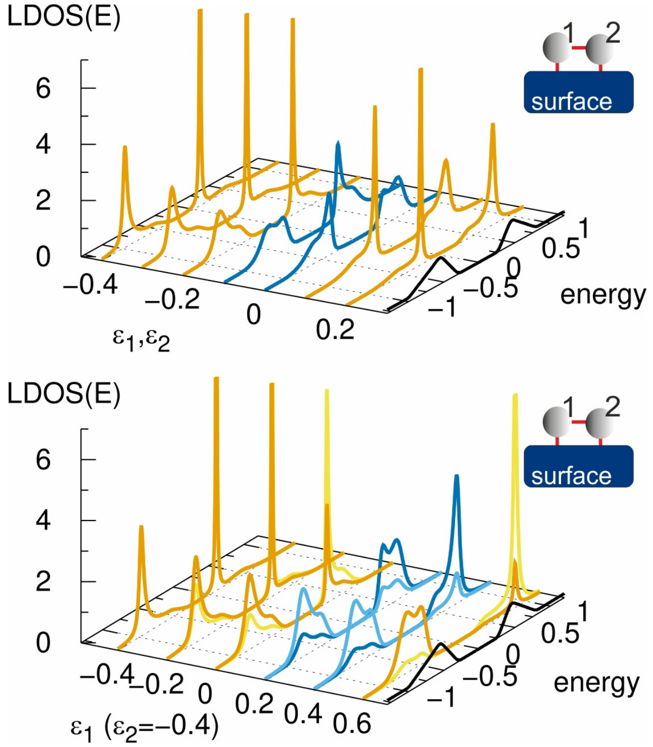

Figure 3: Local DOS as a function of the energy for the system composed of $N = 2$ sites (schematically depicted in the insets) at both atomic sites: $i = 1, 2$ for different on-site energies: $\varepsilon_1 = \varepsilon_2 = -0, 4, -0.3, -0.2, -0.1, 0, 0.1$ and 0.2, respectively (upper panel, LDOS at both sites are the same) and in the bottom panel $\varepsilon_2 = -0.4$ and $\varepsilon_1 = -0.4, -0.2, 0, 0.2, 0.4,$ and 0.6, respectively (LDOS curves at both sites are marked by dark and light shade of colours). Distinctive blue curves are discussed in the text and the black solid lines represent the two-peak DOS of the surface used in the calculations. The hopping integrals $t_{1-2} = 0.5$, $\Gamma_{1,2} = 0.05$.

Short atomic chains, due to the breaking of translational symmetry and the presence of chain ends, exhibit different properties at their edges compared to their center, which can lead to new effects like charge or spin density waves or topological states. As a result, atoms located at the ends of the chain could be defectively characterized by slightly different parameters than those located inside the chain. To analyze this issue for a dimer system,

the bottom panel of Fig. 3 presents the local DOS on both atoms for different values of ε_1 while keeping $\varepsilon_2 = -0.4$ fixed. As seen in most cases, the local DOS curves exhibit strong non-dispersive states (yellow curves), similar to those in the top panel. However, there exists an energy range where the local DOS function is relatively smooth and reflects the features of the substrate DOS (blue curves, bottom panel). In this case, the local DOS on both atoms exhibit up to four peaks. It turns out that such characteristics emerge when the single-particle energy levels of all atoms are located near the peaks of the substrate DOS — in our case, $\varepsilon_1 \simeq +0.4$. Importantly, the local DOS curves for these atoms are not identical — the peak intensities differ significantly (compare the light and dark blue curves in the bottom panel). This indicates that the local DOS functions are asymmetric not only in terms of energy but also spatially. Therefore, the obtained local DOS characteristics for such a dimer, especially in the case of a large difference in on-site energy values within the dimer, do not match the experimental results.

Three-atom system: In Fig. 4, we analyze the local DOS distributions in three-site systems (linear trimers) for two different atomic arrangements, as illustrated in the diagrams. The simplest geometry is a linear arrangement of three sites coupled to the substrate (upper panel). This system exhibits three molecular states, which are clearly visible in the blue curves. The outer atoms display three local DOS peaks (light blue line), while the central atom (dark blue line) exhibits a two-peak local DOS, which is a characteristic feature of a linear three-atom system with one state decoupled from environment. Notably, these states exist within the energy gap of the substrate DOS, making them very sharp and weakly dispersive. For different atomic energy levels of end atoms $\varepsilon_1 = \varepsilon_3$ (ε_2 is constant), some of these states broaden or split if they interact with the peaks of the substrate DOS. However, in all cases, characteristic non-dispersive states can be identified. Since their presence has not been confirmed in the STM differential conductance measurements, we conclude that this particular atomic configuration is not realized on the Si(553)-Au surface.

Topography measurements along short atomic chains indicate a symmetric atomic ar-

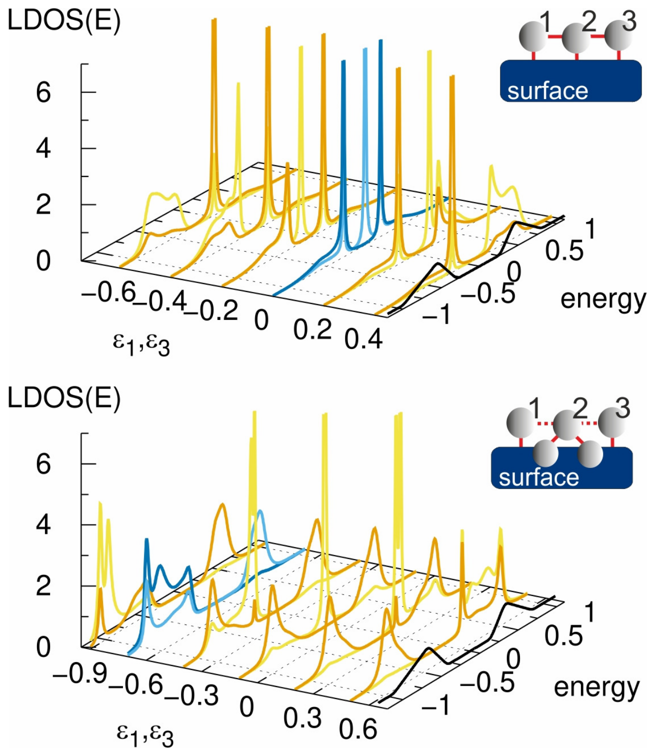

Figure 4: Local DOS for the system composed of $N = 3$ sites in different geometries schematically shown in the diagrams (upper and bottom panel, respectively). The middle onsite energy, $\varepsilon_2 = -0.1$ is constant and the edge sites energies, ε_1 and ε_3, vary from negative to positive values. LDOS at $i = 1, 3$ sites are the same (they are represented by the solid light lines), and LDOS curves for $i = 2$ are marked by the darker lines. Distinctive blue curves are discussed in the text and the black solid lines represent the two-peak DOS of the surface used in the calculations. The hopping integrals $t_{1-2} = t_{2-3} = 0.2$, $t_3 = 0.4$, $\Gamma_i = 0.05$.

rangement in the studied systems. Therefore, in the next step, we investigated the electronic properties of a symmetric three-atom system with a single central site coupled to the substrate via two adjacent atoms from the same terrace. This model effectively describes the properties of a single edge site (Fig. 2 from the main paper). For this geometry and various values of ε_1 and ε_3, the local DOS reveals the presence of nearly non-dispersive bound states (yellow curves). These states typically appear within the energy gap of the substrate DOS or far beyond its main peaks. However, there is a specific range of energy levels of the outer atoms, $\varepsilon_{1/3} \simeq (-0.5, -0.8)$, where the local DOS curves do not exhibit non-dispersive states (blue curves). In this case, the local DOS of the edge sites (1 and 3) is characterized by three peaks at negative energies (within the energy range of the substrate DOS peak) and the absence of states at positive energies. In contrast, the local DOS of the central atom (light blue curve) features only two peaks at negative energies and a single broad state at positive energies. Since such spatially-asymmetrical characteristics were not observed in our experimental measurements of short chains, we conclude that this configuration was not realized in the experiment.

References

1. Blöchl, P. E. Projector augmented-wave method. *Phys. Rev. B* **1994**, *50*, 17953–17979.

2. Perdew, J. P.; Ruzsinszky, A.; Csonka, G. I.; Vydrov, O. A.; Scuseria, G. E.; Constantin, L. A.; Zhou, X.; Burke, K. Restoring the Density-Gradient Expansion for Exchange in Solids and Surfaces. *Phys. Rev. Lett.* **2008**, *100*, 136406.

3. Kresse, G.; Furthmüller, J. Efficient iterative schemes for ab initio total-energy calculations using a plane-wave basis set. *Phys. Rev. B* **1996**, *54*, 11169.

4. Kresse, G.; Joubert, D. From ultrasoft pseudopotentials to the projector augumented-wave method. *Phys. Rev. B* **1999**, *59*, 1758.

5. Schimka, L.; Harl, J.; Kresse, G. Improved hybrid functional for solids: The HSEsol functional. *The Journal of Chemical Physics* **2011**, *134*, 024116.

6. Monkhorst, H. J.; Pack, J. D. Special points for Brillouin-zone integrations. *Phys. Rev. B* **1976**, *13*, 5188.

7. Krawiec, M.; Jałochowski, M. Anisotropic atom diffusion on Si(553)-Au surface. *Phys. Rev. B* **2013**, *87*, 075445.

8. Braun, C.; Gerstmann, U.; Schmidt, W. G. Spin pairing versus spin chains at Si(553)-Au surfaces. *Physical Review B* **2018**, *98*, 121402(R).

9. Yogi, P.; Koch, J.; Sanna, S.; Pfnür, H. Electronic phase transitions in quasi-one-dimensional atomic chains: Au wires on Si(553). *Phys. Rev. B* **2022**, *105*, 235407.

10. Kwapiński, T.; Kohler, S.; Hänggi, P. Electron transport across a quantum wire in the presence of electron leakage to a substrate. *The European Physical Journal B* **2010**, *78*, 75–81.

11. Newns, D.; Read, N. Mean-field theory of intermediate valence/heavy fermion systems. *Advances in Physics* **1987**, *36*, 799–849.

12. Podloucky, R. M. C. Desjonquères and D. Spanjaard: Concepts in surface physics, 2nd edition, Springer Verlag, Berlin Heidelberg, 1996, ISBN-3-450-58622-9, DM 98,00. *Berichte der Bunsengesellschaft für physikalische Chemie* **1997**, *101*, 1081–1082.

13. Kwapiński, T.; Kurzyna, M. Atomic Chains on 2D Hybrid Structures. Materials. *Materials* **2021**, *14*, 3289.

14. da Fonseca, C.; Petronilho, J. Explicit inverses of some tridiagonal matrices. *Linear Algebra Appl.* **2021**, *325*, 7–21.

15. Hu, G.; O'Connell, R. Analytical inversion of symmetric tridiagonal matrices. *J. Phys. A: Math. Gen.* **1996**, *29*, 1511.

16. Kwapiński, T. Conductance oscillations of a quantum wire disturbed by an adatom. *Journal of Physics: Condensed Matter* **2007**, *19*, 176218.
